# A Friend in Need Is a Friend Indeed? Analysis of the Willingness to Share Self-Produced Electricity During a Long-lasting Power Outage

**DOI:** 10.1007/s41471-022-00148-6

**Published:** 2022-12-08

**Authors:** Konstantin Kurz, Carolin Bock, Michèle Knodt, Anna Stöckl

**Affiliations:** 1 Chair of Entrepreneurship, Department of Law and Economics, Technical University of Darmstadt, Hochschulstr. 1, 64289 Darmstadt, Germany; 2 Institute of Political Science, Department of History and Social Sciences, Technical University of Darmstadt, Landwehrstraße 50A, 64293 Darmstadt, Germany

**Keywords:** Energy Resilience, Social Dilemma, Prosocial Behaviour, PV System, Power Outage, D64, D91, H12, H41, L94, Q42, Q48

## Abstract

Will private households owning a photovoltaic system share their electricity during a long-lasting power outage? Prior research has shown that our energy systems need to become more resilient by using dispersed energy sources—a role that could well be performed by these private photovoltaic systems, but only if their owners decide to share the produced electricity, and not consume it themselves. Considering the potential of this approach, it is indispensable to better understand incentives and motives that facilitate such cooperative behaviour. Drawing on theories of social dilemmas as well as prosocial behaviour, we hypothesize that both, structural solutions such as increased rewards as well as individual motives such as empathy-elicited altruism and norms predict cooperation. We test these hypotheses against a dataset of 80 households in Germany which were asked about their sharing behaviour towards four different recipient groups. We show that the effectiveness of motives differs significantly across recipient groups: Individual (intrinsic) motivations such as empathy-elicited altruism and altruistic norms serve as a strong predictor for cooperative behaviour towards related recipients as well as critical infrastructure, whereas higher rewards partially even reduce cooperation depending on the donor’s social value orientation. For the recipient groups neighbours and public infrastructure, no significant effect for any of the tested incentives is found. Contributing to literature on social dilemmas and energy resilience, these results demonstrate the relevance of individual rather than structural incentives for electricity sharing during a power outage to render our energy provision more resilient. Practical implications for policymakers are given.

## Introduction

Energy experts raise growing concern that the ever-increasing number of natural disasters (Williams et al. 2017), the rising share of renewable energies (Bialek 2020), and the challenges due to the current energy crisis pose significant threats for the stability of the electricity networks (Lovins and Lovins 1982; United Nations 2016; Chaudry et al. 2009). The consequences of long-lasting power outages^1^ will be detrimental, threatening the supply of basic needs such as water, food, healthcare, security, or food (Petermann et al. 2010). As a response to this challenge, societies are increasingly concerned with energy resilience, defined as the capacity of an energy system to recover from external disturbances and maintain a sufficient level of service (Kishita et al. 2017; Roege et al. 2014).

Experts see one very promising approach for achieving such a sufficient level of service during a disturbance in the use of the continuously growing number of photovoltaic (PV) systems combined with battery storage in order to increase the resilience of our power network (Hirschl et al. 2018; Perez et al. 1997, 2004). More precisely, when the national or regional electricity supply fails, citizens who have a PV system feed the generated energy into the local grid and thus make the entire system more resilient (Lovins and Lovins 1982). However, this approach will only work if the owners of the PV systems agree to share at least part of their energy resources, and not consume them themselves. This circumstance serves as a classic example of a social dilemma, i.e. a situation in which the individual receives a higher payoff for not cooperating, but all individuals collectively will receive a lower payoff if all choose this strategy (Dawes 1980).

Social dilemmas related to energy sharing are not a new phenomenon. Consider the case of the small Dutch village Huizinge from 1979. Due to extraordinarily heavy snowfall, Huizinge was not reachable and there was a complete power outage (Liebrand 1983). However, one of the 150 residents had a generator that could provide enough energy for all the people in the community, but only if they significantly cut their energy consumption (Van Lange et al. 2013). Unfortunately, most people did not restrain themselves sufficiently, leading to a collapse of the generator and as such also the electricity supply (Van Lange et al. 2013). Anecdotal observations like this Huizinge case inevitably raise the question of how to achieve a sufficient level of cooperation to enable PV-based emergency power supply.

Prior research has not presented a clear answer to solving such a social dilemma. Solutions to foster this intended cooperative behaviour are generally divided into structural and individual (motivational) approaches (Messick and Brewer 1983). Structural strategies try to incentivize cooperation by changing the payoffs for the individual (e.g. by increasing feed-in tariffs) such that it is no longer a social dilemma (Dawes 1980). In contrast, individual solutions aim to arouse people’s intrinsic motivation to act cooperatively, leveraging individual-level motives such as empathy-elicited altruism (Batson 1991) or altruistic norms (Schwartz 1977) that let people engage in prosocial action even if this reduces their individual payoffs (Bierhoff 2002; Van Lange et al. 2013; Penner et al. 2005). Interestingly, research has shown that structurally introducing (financial) rewards can convert social decisions into business decisions, thus potentially crowding out pre-existing selfless motives (Frey and Oberholzer-Gee 1997; Deci and Ryan 2000). Taking these ambiguous insights together, prior research does not provide clear evidence on which of the two solutions is more effective for motivating cooperative behaviour during a power outage.

We address this gap by developing a model of social dilemma acting during a power outage, combining theoretical concepts from research on energy resilience (Kishita et al. 2017; Chaudry et al. 2009), social dilemma (Van Lange et al. 2013; Dawes 1980), and pro-social behaviour (Bierhoff 2002; Penner et al. 2005). We hypothesize that both, structural solutions such as increased rewards and individual solutions such as empathy-elicited altruism (Batson 1991), reciprocal motives (Trivers 1971) and altruistic norms (Schwartz 1977) increase the propensity to act cooperatively. We empirically validate our hypotheses using a survey among 80 inhabitants from a medium-sized German municipality. As part of the survey, participants were asked to indicate their willingness to share electricity with the four different stakeholder groups neighbours, friends and family, public non-critical infrastructure, and critical infrastructure. Our results show that the influence of the motives differs significantly across stakeholder groups. Concerning sharing with friends and family as well as critical infrastructure, individual factors such as empathy-elicited altruism (Batson 1991) and altruistic norms (Schwartz 1977) show a moderate to strong positive effect on the willingness to share, whereas structural rewards increase cooperation only among self-oriented individuals, but had no or even a negative effect among others-oriented participants (Messick and McClintock 1968; Frey and Jegen 2001). None of the tested motives showed a significant effect on the sharing behaviour towards neighbours and public non-critical infrastructure. These findings coincide with a generally lower willingness to share found for both recipient groups.

With these results, our work significantly advances our understanding of energy resilience (Kishita et al. 2017). While prior research unanimously agrees on the need to use more dispersed energy sources such as private PV systems (Lovins and Lovins 1982; Hirschl et al. 2018), this study is the first to investigate the sharing willingness of PV system owners as a necessary pre-condition. By showing the relevance of intrinsic motivational factors for the decision to act cooperatively during a power outage, this work makes a strong contribution to our understanding of cooperative behaviour of private households during times of adversity, and the feasibility of implementing the residential PV system approach in practice the feasibility of implementing the residential PV system approach in practice. We hence advance our knowledge on prosocial behaviour on the so-called ‘meso level’—on conditions under which one person helps another person—and on the ‘micro level’ by shedding light on the individual motives that contribute to energy sharing behaviour (Penner et al. 2005). In doing so, our study supports the view raised in recent research that social-ecological resilience must to be supported by adaptive governance measures that successfully integrate individual views for increasing community resilience (Folke 2006). We thereby also answer calls for further research on energy transition in business research (Bichler et al. 2022).

Second, we also contribute to literature on social dilemmas (Dawes 1980). To the best of our knowledge, no prior work has investigated the motives for cooperative acting in the social dilemma of electricity sharing during a power outage, which is a necessary prerequisite for shedding light on the question where to position levers to increase the possibility to use privately-owned PV systems to increase energy resilience of our society. We show that, even though the influence of motives significantly differs among recipient groups, the social dilemma of electricity sharing appears to rather be a game of individual intrinsic motives than incentive-based structures (Messick and Brewer 1983). As such, these results provide clear practical implications, indicating that policymakers should place more emphasis on fostering strong community ties (Van Vugt 2002; Puddifoot 1995) than on higher feed-in tariffs.

## Theory and Hypotheses

### Energy Resilience

The concept of resilience originates from ecosystem theory and describes the capacity to “bounce back”, i.e. the tolerance of a system to endure disturbances (Walker 2020; Zolli and Healy 2012). The main feature of the resilience concept is the idea that the “survivability” of a system is not based on achieving static states of equilibrium and sealing itself off from impacts. On the contrary, a system is considered to be resilient if it reacts dynamically and with adaptive capacity to vulnerability, i.e. has a temporally and spatially elastic response to crises (Engels 2018). Further detailing this dynamic system, Maguire and Hagan (2007) distinguish three dimensions: (1) The resilience of a system to take precautions against shocks or gradual changes that have occurred (“resilience”); (2) The capacity to restore the initial state relatively quickly and to react creatively to disruptive events and absorb them (“recovery”); and (3) the ability of a system to achieve the highest possible level of function and protection by adapting to changing conditions (“creativity”). In other words, resilience also covers the dimension of preparedness, describing flexible reactions to anticipated disruptions and the ability to “bounce forward” (Engels et al. 2018, p. 52). Overall, resilience is thus often considered a process-like ability (Folke 2006).

Driven by the increasing relevance of natural disasters as well as the rise of volatile renewable energy sources (United Nations 2016; Lovins and Lovins 1982), literature has increasingly applied these concepts to the context of energy networks (Kishita et al. 2017; O’Brien and Hope 2010; Roege et al. 2014; Gatto and Drago 2020). Following Kishita et al. (2017), we define energy resilience as the capacity of an energy system “to speedily recover from external shocks and to provide alternative means of maintaining an acceptable level of services to consumers in the event of external disturbances” (p. 115). In their famous “Brittle Power” book, Lovins and Lovins (1982) suggest dispersed diverse energy sources as alternative means to increase energy resilience. Residential PV systems and battery storage could take on this alternative means role (Hirschl et al. 2018; Perez et al. 1997, 2004). If prepared, they can feed in the produced electricity during a power outage, and thus supply (certain) members of a society with electricity. Theoretically, the energy system would thus be able to provide some “acceptable level of services to consumers” (Kishita et al. 2017, p. 115), i.e. be resilient (at least to a certain degree, acknowledging that the amount of energy currently produced will be lower compared to non-crisis supply; Perez et al. 2004).

For practical implementation, there remain two challenges to be solved: First, from a technical perspective, it requires the installation of inverters operating in the so-called *grid-forming* mode (Rocabert et al. 2012; Rosso et al. 2021). Currently, inverters can only operate in the *grid-supporting* mode (Zeb et al. 2018), continuously measuring the grid’s voltage to supply energy at the right time. If no voltage can be measured (e.g. in case of a power outage), the inverter is shut offline. In contrast, inverters that are also able to operate in a *grid-forming* mode provide voltage to the grid independently of the external situation, and can thus be used to supply power to the grid also during a power outage (Knodt et al. 2022).

Second, and being the core of this work, it requires PV system owners to share their resources (to cooperate) with related or unrelated others, and not consume their energy themselves. It is obvious that (in the short-term) PV system owners are better off when consuming their energy themselves since it increases their personal utility by allowing them to fulfil their daily basic needs (Van Lange et al. 2013). However, if all PV system owners choose not to share (i.e. all defect), all will (in the long-term) be worse off than if all cooperated, since the then persisting breakdown of critical infrastructure and associated cascading effects will reduce the individuals’ personal utility and even threaten their own survival (Petermann et al. 2010; Lovins and Lovins 1982; Knodt et al. 2022). This situation perfectly describes a social dilemma (Dawes 1980), and the willingness to cooperate in such a situation has, to the best of our knowledge, not been researched yet. With the aim to fill this gap, the following section will proceed with a more detailed definition of this social dilemma concept.

### Social Dilemmas and Public Goods

We follow the definition of Dawes (1980) and define a social dilemma as a situation in which “(a) the […] payoff to each individual for defecting behaviour is higher than for cooperative behaviour” and “(b) all individuals in the society receive a lower payoff if all defect than if all cooperate” (p. 170). In such situations, the (a) higher individual payoff is often more short-term while the (b) collective consequences are long-term (Van Lange et al. 2013). Social dilemmas frequently occur in situations related to the contribution and sharing of public goods (Fehr and Gächter 2002). In a typical public goods dilemma, group members can benefit from a good without contributing to its provision, leading to the risk that the good will be depleted or not even provided at all (Van Dijk and Wilke 1995). This circumstance became widely known as “The Tragedy of the Commons” after an impactful article from Hardin (1968). Hardin (1968) argued that if a resource is at free disposal of individuals, all citizens try to generate the highest possible yield for themselves in order to maximize their own utility rationally. If the resource is exhausted, the consequences of depletion must be borne by all citizens. This tragedy of the commons means that free access to a finite resource will necessarily lead to its overexploitation due to the rational decisions of all individuals (Hardin 1968).

After observing multiple examples of continuous use of public goods without their depletion from all around the world, Ostrom (1990) later countered that self-organizing forms of local collective actions could serve as an effective mean for overcoming the tragedy of the commons. Her approach argues that if societies have the freedom to coordinate themselves when using a public good (Cox 2019), their citizens do not act selfishly but try to develop a suitable solution strategy together (Ostrom 1990), i.e. act cooperatively. Cooperative action requires some design principles to be organized and maintained, such as clearly defined thresholds of acceptable use per individual, participation in decision-making, effective monitoring, or graduated sanctions (Ostrom 1990). With these design principles, citizens can establish a suitable framework as the basis for a collective sharing strategy (Cox 2019).

The subsequent section will transfer these findings to the social dilemma of electricity sharing. In this dilemma, the energy produced by the privately-owned PV system represents the public good that the PV system owners could contribute to the society (Van Dijk and Wilke 1995).

### A Model of Incentives for Cooperative Acting During Power Outages

The intended decentralized resilience system hinges on the willingness of residential PV system owners to contribute their energy, i.e. choose the *cooperation* strategy (Dawes 1980; Van Dijk and Wilke 1995). As indicated, solutions to foster such cooperative behaviour are usually differentiated into structural and individual (motivational) approaches (Messick and Brewer 1983). While structural solutions try to encourage cooperation through changing the actual payoffs in a dilemma situation, individual solutions aim to leverage and arouse pre-existing personal motives for prosocial behaviour (Van Lange et al. 2013).

Individual solutions are built on the premise that individuals sometimes act on not fully self-interested motives (Van Lange et al. 2013), a long-neglected notion. From a social psychology perspective, the dominant view among Western philosophers such as Aristotle, Thomas Hobbes, Friedrich Nietzsche, or Sigmund Freud was that people are exclusively self-interested (Bierhoff 2002; Batson 1991). This view was only rarely challenged until the outcomes of several experiments led Batson (1991) to assert the existence of an independent altruistic system. Incorporating this new theoretical stream, current psychology literature often suggests three broad classes of motives for social behaviour: Self-interest, internalized norms and principles, and empathy (Bierhoff 2002). Whereas broader views on prosocial behaviour define it as help which does not have its roots in professional obligations (Bierhoff 2009) or acts that significant parts of the society would declare as being beneficial to others (Penner et al. 2005), we consider a specific case of prosocial behaviour in which the selfless decision of an individual towards their direct community is considered to increase the resilient power of this community. We hence consider prosocial behaviour of individuals who offer situational contribution through direct action in case of adversity.

Selfless motives are also discussed in socio-biology research, which, in contrast to socio-psychologists, adopts an outcome view for explaining prosocial motives. Prominent theories here are the inclusive fitness theory (Hamilton 1964), reciprocal altruism (Trivers 1971), and the group selection theory (Sober and Wilson 1999). Most of these theories follow the general conception of neo-Darwinian models which define evolutionary success as the survival of an individual’s genes and argue that humans acting prosocially following genetically-based predispositions are more likely to achieve this outcome (Penner et al. 2005).

Building on these theories, our model to explain and influence the degree of cooperation in the context of increasing the resilience of our energy system, aims to capture both, structural influences that refer to self-interested motives of PV system owners, and individual solutions incentivizing selfless motives driven by the presented theories. Overall, we expect that the level of cooperation is influenced by (1) structural changes such as rewards (Piliavin et al. 1981), (2) people’s level of empathy (Batson 1991), (3) the degree to which they have experienced receiving selfless help (Trivers 1971), and, finally, (4) the degree to which people are aware of the outage’s negative consequences for others, thus letting them act due to an altruistic personal norm (Schwartz 1977).

#### Rewards as Structural Changes

The concept of imposing structural changes follows the traditional view that people behave rationally and are universally motivated by self-interest. Hence, individuals will only act prosocially if the rewards of such behaviour exceed its costs (Penner et al. 2005). This understanding is conceptualized by the cost-reward model developed by Piliavin et al. (1981). According to the model, the bystanders’ intervention in case of an emergency depends on the costs and rewards of helping versus those of not helping (Piliavin et al. 1981; Bierhoff 2002). Put simply, bystanders will help if they expect that this action will yield the best personal outcome for them (Penner et al. 2005; Dovidio et al. 1991). A fundamental notion of the model is the understanding that rewards and costs not only refer to directly measurable financial results, effort, or time loss, but also to psychological outcomes such as negative empathic arousal when seeing others suffer, guilt, or self-esteem damage (Dovidio et al. 1991; Schroeder et al. 1995).

This approach received a lot of empirical confirmation (Clark 1976; Gaertner and Dovidio 1977). Numerous experiments provide evidence that high costs of helping reduce the willingness to help (Gross et al. 1975; Darley and Batson 1973; Fritzsche et al. 2000), and that high costs of not helping increase cooperation behaviour (Deaux 1974; Bickman and Kamzan 1973; Bierhoff 2002). Likewise, rewards were found to influence the net-benefit calculation in favour of cooperation (Van Lange et al. 2013; Parks 2000; Rand et al. 2009; Rapoport et al. 1965). In the context of social dilemmas, a recent meta-analysis based on 187 effect sizes demonstrated that offering rewards has a moderately positive effect on cooperation (Balliet et al. 2011). Rewards structurally change the payoff structure (Messick and Brewer 1983), and thus reduce the discrepancy between self- and collective interest (Dawes 1980).

Based on this evidence, we argue that the willingness of PV system owners to share their electricity should increase if they receive additional rewards for this behaviour. A straightforward approach for such rewards would be an increase of the feed-in remuneration during a power outage, such that the money received per kilowatt-hour during adversity is a multiple of the value during regular times. Such an increase would structurally change the dilemma’s payoffs, in that cooperative behaviour becomes financially more attractive and thus also increases the individual’s own utility (Penner et al. 2005) as well as the resilience of the whole energy system. Transferring the positive effects of incentives found in other social dilemmas, we postulate the following hypothesis:

##### Hypothesis 1a

*Structurally changing the rewards—by offering an increased feed-in remuneration during a power outage—increases people’s willingness to share electricity during that outage.*

There is, however, an ongoing discussion whether reward schemes are equally effective among people with different value orientations (Van Lange et al. 2013). Drawing on social value orientation theory (Messick and McClintock 1968), several scholars argue that rewards can undermine intrinsic motivation to cooperate and true motives to do good, especially among others-oriented individuals (Deci and Ryan 2000; Deci et al. 1999; Bénabou and Tirole 2006). The explanation for this notion is straightforward: If an others-oriented individual derives value by performing cooperative actions, paying this person for this behaviour reduces her opportunity to indulge in truly selfless feelings (Frey and Oberholzer-Gee 1997; Frey 1994). Indeed, studies show that financial incentives can transform ethical issues into a business decision (Gneezy and Rustichini 2000; Tenbrunsel and Messick 1999). Social psychologists have therefore argue that there are hidden costs of rewards (Lepper and Greene 1978) leading to a crowding out of intrinsic motives (Frey and Oberholzer-Gee 1997). These effects were found to be particularly strong among people with pre-existing intrinsic motives (Eckel et al. 2005; Hossain and Li 2013; Van Lange et al. 2013) and those who feel strongly connected to their community (Van Vugt 2001; Frey and Oberholzer-Gee 1997). Transferring these findings to the social dilemma of electricity sharing, we expect that—in contrast to the hypothesized positive main effect—offering rewards will negatively affect the willingness to cooperate among PV system owners with a high level of others-orientation (i.e. pre-existing intrinsic motivation).

##### Hypothesis 1b

*The level of others-orientation moderates the relationship between rewards and willingness to share, such that rewards will be less effective among others-oriented individuals.*

#### Empathy-Elicited Altruism

Besides structural changes, individual factors can be responsible why people show prosocial behaviour (Bierhoff 2009; Van Lange et al. 2013). Building on the socio-psychologic empathy-altruism hypothesis, we expect that people’s willingness to share electricity will be higher when they experience of empathy-elicited altruism. The notion that there is an altruistic system influencing prosocial behaviour was coined by Batson (1991) who showed in experiments that people act on empathy-elicited altruistic motives. He summarized these findings in the empathy-altruism hypothesis (Batson 1991). The underlying idea of this hypothesis is that if an individual with a high level of dispositional empathy observes another person in need, this individual will respond with altruistic behaviour with the aim to reduce the other’s suffering (Batson 1991; Bierhoff 2002). Escaping the situation is not an option for the high-empathy individual, since the altruistic motivation will prevail until the victim receives help either by the observer or by a third person (Bierhoff 2002; Batson et al. 1988). This conception was confirmed across various experiments (Bierhoff 2002; Batson et al. 2015), and is additionally supported by several meta-analytic results showing that dispositional empathy is associated with prosocial behaviour (Davis et al. 1999; Penner et al. 2005; Eisenberg and Miller 1987). For instance, Batson and Ahmad (2001) showed that in the high-empathy group, nearly 45% of participants made a cooperative choice, compared to only roughly 5% in the low-empathy group. Transferring these findings to the social dilemma of electricity sharing, we expect that altruistic motives elicited by empathy increase an individual’s propensity to act cooperatively (i.e. share).

##### Hypothesis 2

*Empathy-elicited altruism increases an individual’s willingness to share electricity.*

#### Direct Reciprocal Altruism

A socio-biologic approach to explain motives for prosocial behaviour towards unrelated victims is the direct reciprocal altruism theory (Penner et al. 2005). Following this theory, we expect that the willingness to share electricity will be higher among those individuals that have received help previously (Van Lange et al. 2013). The theory was developed by Trivers (1971) who argues that if humans behave altruistically, they will receive a payback later. This norm of reciprocity was already propagated by the Roman statesman and philosopher Cicero, who said that “there is no duty more indispensable than that of returning kindness” (Gouldner 1960, p. 161). Direct reciprocity refers to a narrow strategy to act prosocially only towards those that have helped in the past (Axelrod and Hamilton 1984; Trivers 1971; Nowak and Sigmund 2005). Indeed, studies show that people are more likely to help individuals that have offered them help before (Berg et al. 1995; Falk 2007; Wilke and Lanzetta 1970). The reciprocity theory also resembles the *Tit-For-Tat* approach, which is found to produce greater payoffs than any other strategy in the famous prisoner’s dilemma game (Axelrod and Hamilton 1984). Further (indirect) evidence for the reciprocal altruism theory can be transferred from the universal existence of the reciprocity norm in nearly every culture around the world (Penner et al. 2005; Schroeder et al. 1995; Komter 1996). Building on this strong evidence, we expect that the willingness to share electricity in times of power outages will be higher when the donor has received help before.

##### Hypothesis 3

*The willingness to share electricity will be higher among those individuals that have received help before.*

#### Perceived Consequences & Altruistic Norm

Our final individual factor for explaining prosocial behaviour builds on the norm activation theory developed by Schwartz (1977). Following his concept, we expect that the degree to which people perceive the crisis as a threat increases their willingness to share electricity.

The norm activation theory posits that for an individual to behave altruistically, this individual must be (1) aware of the negative consequences of a certain situation for others, and (2) feel both, responsible and able to prevent this state (Schwartz 1977). When both conditions are fulfilled, people develop an altruistic personal norm to engage in prosocial behaviour (Guagnano et al. 1995).

Evidence for the validity of this theoretical concept has been found in several studies and situations. For instance, Black et al. (1985) showed that a personal norm for household energy efficiency activated by the awareness of consequences of such efficiency for others increases an individual’s willingness to invest in low-cost efficiency improvements. Furthermore, a meta-analysis on 17 studies from the field of environmental behaviour showed that the more people possess knowledge about environmental issues (i.e. are aware of negative consequences), the more they are likely to engage in responsible (altruistic) environmental behaviour (Hines et al. 1987; also see Kalkbrenner and Roosen 2016). Extending the basic model from Schwartz (1977), Stern et al. (1993) demonstrated that altruistic actions are not only activated by perceived negative consequences for others, but also for oneself. Transferring these findings to our examined social dilemma of electricity sharing during a power outage suggests that altruistic behaviour should be particularly dominant among individuals that perceive the crisis as particularly threatening, potentially causing a negative state for themselves and others. In line with this theorizing, a recent study on the COVID-19 pandemic showed that the more people perceived the disease as a personal threat, the more they behaved altruistically (Vieira et al. 2020). Summarizing these arguments, we thus postulate the following hypothesis:

##### Hypothesis 4

*The more individuals perceive the crisis as a threat to themselves and others, the higher will be their willingness to share their electricity with these others.*

## Methodology

### Dataset

#### Research Setting

We used a survey to empirically test our hypotheses. The survey was conducted among citizens in districts of Darmstadt, a relatively affluent medium-sized city in Germany. The districts were chosen due to their relatively high share of PV system owners. The survey was launched in March 2021 and ended in April 2021. A total of 408 printed questionnaires were manually distributed to residents in the mentioned respective districts. Of these, 127 questionnaires were returned by mail, representing a still acceptable response rate of approximately 31% (Baruch and Holtom 2008). Of the returned questionnaires, roughly 69% were completed by men, 30% by women, and 1% respondent of diverse gender. The age groups represented in the sample varied from 0–18 (2%), 19–29 (2%), 30–49 (20%), 50–69 (52%), and older than 70 (24%). The typical respondent lived in a detached single-family house (61%) and had a photovoltaic or solar thermal system installed (65%). The homeownership rate was 87%. The average household size was roughly 2.8 residents. The data was then digitized and cleaned according to scientific standards. We removed missing values following a listwise deletion approach as the most common and conservative solution for treating missing values (Acock 2005; Tsikriktsis 2005). After doing so, our final sample consisted of 80 observations.

#### Questionnaire

The questionnaire consisted of four parts. The first part included demographic questions as well as questions on the participants’ housing situation. The second part asked questions about previous experience with crises and power outages. The third section covered questions on the behaviour during a power outage situation. Participants were asked to imagine the scenario of a 24 h large-scale blackout (Petermann et al. 2010). They were also told to assume that they would possess a PV system which they could decouple from the usual grid to either consume the produced energy themselves or to share it with certain stakeholders. The fourth section contained questions on respondents’ political attitude as well as participation in communal political activities. Following best practice recommendations (Hunt et al. 1982), the quality of the questionnaire was tested in a standard pretest to ensure comprehensibility and clarity of the questions and answer options. Possible weaknesses were corrected after the pretest. The pretest resulted in a response time of approximately 20 min.

### Measures

#### Dependent Variables

##### Electricity Sharing

The willingness to share electricity during a power outage was assessed separately for four different stakeholder groups: (1) Neighbours, (2) Friends & Family, (3) Public infrastructure such as schools, sports facilities, universities, or community centres, and (4) critical infrastructure such as hospitals, police, communication network providers, or water suppliers. For each stakeholder group, respondents had to indicate on a four-point scale the degree to which they would share their electricity with that group, ranging from 1 = *I would not share any electricity*, over 2 = *I would share excess electricity (my own consumption is not restricted)*, 3 = *I would share parts of my electricity (my own consumption is restricted)*, to 4 = *I would share all of my electricity*. Investigating each recipient group separately allows for a more detailed analysis of the effectiveness of the hypothesized incentives on the willingness to share with different parts of the society. Using these scales, four dependent variables (one for each stakeholder group) with values from 1 to 4 were created (*Electricity_Sharing*).

#### Independent Variables

##### Feed-In Remuneration

We operationalized feed-in remuneration through a single item measure, asking respondents to indicate how much Euro (EUR) per kilowatt-hour (kWh) they would deem appropriate for sharing electricity during a power outage. A regular value of 0.08 EUR/kWh was mentioned as baseline reference (regular value from 2020; Fraunhofer ISE 2022). Participants were given five answer options with different increases compared to this baseline level, ranging from *No increase* to *More than 1* *EUR*. The answers were then transferred into an ordinal scale ranging from 1 to 5, with 1 = *No increase *and 5 = *More than 1* *EUR*, as input for our *Feed-In *variable.

##### Others-Orientation

The degree of others-orientation was operationalized by an item asking respondents about the main motives for the installation of a PV system. If participants already possessed a PV system, they were required to mention the motives they had when installing it. Those who did not have one yet were asked to state the motives they *would* have when installing one. Previous studies have shown that a prosocial value orientation is positively associated with environmental and climate protection behaviour (Cameron et al. 1998; Joireman et al. 2001; Van Lange et al. 2013). Transferring these results, we thus argue that the motivation to engage in climate protection thus serves as a suitable proxy for the degree of others-orientation. Leveraging this reasoning, respondents were given four response options, including self-oriented motives such as (1) *saving energy costs*, (2) *autonomous energy supply*, and (3) *expected value increase of property*, and the other-oriented motive (4) *contribution to renewable energies and climate protection*. Our variable *Others-Orientation* thus equals 1 if the main motive for installing a PV system was or would be climate protection.

##### Empathic Altruism

We measure participants’ level of empathy-elicited altruism by the degree to which they proactively engaged in helping behaviour during the COVID-19 pandemic. The pandemic caused strong negative consequences for humans globally, including direct health impacts as well as indirect impacts resulting from non-pharmaceutical prevention measures (WHO 2022; Perra 2021). At the same time, the crisis was also characterized by various forms of prosocial behaviour (Haesler et al. 2021). Studies showed that a significant motive for such altruistic acting was empathy (Christner et al. 2020; Pfattheicher et al. 2020). Using this fact, respondents were asked whether they had voluntarily helped different stakeholders during the pandemic, including neighbours, friends, relatives, public institutions, or charity organizations. Our variable *Help_Given* thus equals 1 if respondents replied that they had helped any of these stakeholder groups.

##### Reciprocity

The assessment of reciprocal motives was measured using respondents’ answers on whether they had received help during the COVID-19 pandemic. As outlined above, the crisis had a detrimental impact on the personal well-being of many people (WHO 2022). Partly, individuals could not manage their lives on their own anymore, and thus received help from related or unrelated others (Haesler et al. 2021). Leveraging this circumstance, we created a variable *Help_Received* equalling 1 if the respondent had received help during the COVID-19 pandemic as a measure for the hypothesized reciprocal motives (Trivers 1971).

##### Perceived Threat

We measure the perceived threat and expected consequences of the power outage by the degree to which respondents are willing to take investments in crisis preparation measures. The willingness to invest resources was shown to be positively related to the degree an individual considers an event to be detrimental (Hansla et al. 2008; Obeng and Aguilar 2018). Against that background, respondents were asked to indicate how much they would generally invest to prepare for the described scenario of a long-lasting power outage. The response scale ranged from 1 = *0* *EUR* to 4 = More than *5000* *EUR*, with 2 = *500–1000* *EUR* and 3 = 1001–5000 EUR. We used this ordinal scale for our *Investment_Readiness* variable.

#### Control Variables

Following best practice guidelines, we included control variables we expected to correlate with our dependent or independent variables based on prior theoretical and empirical research (Bono and McNamara 2011). First, we used an ordinal scale to control for the respondents’ *Age*, given that previous research found age to influence both, the behaviour in social dilemmas (McClintock and Moskowitz 1976; Van Lange et al. 2013) as well as the level of prosocial action (Van Lange et al. 1997; Bierhoff 2002). Respondents could indicate their age using given brackets, with 1 = *Younger than 18*, 2 = *19–29*, 3 = *30–49*, 4 = *50–69*, and 5 = *More than 70*. Next, we accounted for potential influence of *Gender*^2^, as research has likewise shown that women behave differently in compete-versus-cooperate situations (Van Vugt et al. 2007), and also differ in their level of empathy and as such expected prosocial behaviour (Eisenberg and Lennon 1983; Bierhoff 2002). As a context-specific control variable, we included a measure indicating whether a respondent had already experienced a power outage (*Outage_Experience*). The experience of such a crisis could likely influence the willingness to share, either negatively by showing what it means to be without electricity for quite some time, or positively by outlining the value of a functioning critical infrastructure (Rubin and Rogers 2019). We then also included the *Household_Size* as a further context-specific control, as it is strongly associated with the own consumption needs (Van Vugt 2001), which may in turn influence the willingness to share. Finally, we controlled for the *Household_Income* as a proxy for the respondent’s social status, since prior studies showed that people’s social status influences their level of prosocial behaviour (Bierhoff 2002; Piff et al. 2010). Household income was measured using a three-point scale covering *0–2000* *EUR, 2000–5000* *EUR*, and *more than 5000* *EUR*.

## Results

Table 1 provides the descriptive statistics and correlations for all variables. An intriguing descriptive result can already be observed when comparing the means across the four dependent variables: The mean willingness to share electricity is highest towards critical infrastructure (2.69), followed by friends and family (2.58), neighbours (2.46), and lowest for public non-critical infrastructure (1.99). Noticeable is also the lower standard deviation for the sharing behaviour towards neighbours, where no respondent indicated the willingness to share all available electricity.

**Table 1:** Descriptive Statistics and Correlations

	Mean	S.D.	Min	Max	1	2	3	4	5	6	7	8	9	10	11	12	13	14	15
1. Electricity_Sharing_Neighbours	2.46	0.76	1.00	3.00	1	–	–	–	–	–	–	–	–	–	–	–	–	–	–
2. Electricity_Sharing_Friends_Family	2.58	0.85	1.00	4.00	0.54	1	–	–	–	–	–	–	–	–	–	–	–	–	–
3. Electricity_Sharing_Public_Infra	1.99	0.85	1.00	4.00	0.36	0.39	1	–	–	–	–	–	–	–	–	–	–	–	–
4. Electricity_Sharing_Critical_Infra	2.69	0.91	1.00	4.00	0.10	0.38	0.50	1	–	–	–	–	–	–	–	–	–	–	–
5. Feed-In	2.11	1.15	1.00	5.00	0.01	0.17	−0.10	−0.05	1	–	–	–	–	–	–	–	–	–	–
6. Others-Orientation	0.83	0.38	0.00	1.00	0.15	0.23	0.23	0.06	−0.13	1	–	–	–	–	–	–	–	–	–
7. Help_Given	0.60	0.49	0.00	1.00	0.06	0.43	0.14	0.25	−0.08	0.16	1	–	–	–	–	–	–	–	–
8. Help_Received	0.08	0.27	0.00	1.00	−0.30	−0.19	−0.16	−0.01	−0.03	−0.12	−0.06	1	–	–	–	–	–	–	–
9. Investment_Readiness	2.65	0.95	1.00	4.00	0.05	0.19	0.12	0.26	0.03	0.11	−0.03	0.01	1	–	–	–	–	–	–
10. Age	3.80	0.88	1.00	5.00	−0.11	−0.35	−0.26	−0.44	−0.17	−0.11	−0.19	0.23	−0.10	1	–	–	–	–	–
11. Gender (Female)	0.29	0.46	0.00	1.00	−0.21	−0.11	−0.06	0.10	0.11	−0.07	0.07	0.34	−0.20	−0.11	1	–	–	–	–
12. Outage_Experience	0.73	0.45	0.00	1.00	−0.25	−0.14	−0.04	−0.03	0.01	0.08	−0.05	0.07	−0.05	0.15	0.08	1	–	–	–
13. Household_Size	2.80	1.30	1.00	6.00	0.15	0.20	0.25	0.25	0.15	0.01	−0.05	−0.10	0.07	−0.41	−0.14	0.03	1	–	–
14. Household_Income_2001–5000	0.49	0.50	0.00	1.00	−0.13	−0.07	−0.02	0.06	−0.21	−0.14	−0.22	0.10	−0.04	0.05	−0.01	−0.18	−0.10	1	–
15. Household_Income_> 5000	0.48	0.50	0.00	1.00	0.21	0.18	0.04	0.05	0.21	0.11	0.27	−0.18	0.03	−0.10	−0.05	0.14	0.21	−0.93	1

To statistically test our hypotheses, we conducted eight separate ordinary-least squares (OLS) regressions, two for each stakeholder group to enable a separate analysis of the hypothesized moderation effect of others-orientation on feed-in remuneration, respectively. To cope with heteroscedasticity (Hayes and Cai 2007), we implemented robust standard errors. Table 2 reports regression coefficients, the standard errors and their levels of significance for all variables.

**Table 2:** Regression Results

	Neighbours		Friends & Family		Public Infrastructure		Critical Infrastructure	
	Model 1	Model 2	Model 3	Model 4	Model 5	Model 6	Model 7	Model 8
	Electricity Sharing	Electricity Sharing	Electricity Sharing	Electricity Sharing	Electricity Sharing	Electricity Sharing	Electricity Sharing	Electricity Sharing
*Constant*	1.912*	1.806	0.954	0.001	1.638*	0.769	1.964**	0.796
	(0.995)	(1.103)	(0.609)	(0.748)	(0.829)	(0.894)	(0.769)	(0.760)
*Control Variables*								
Age	0.037	0.039	−0.153*	−0.135	−0.107	−0.091	−0.382***	−0.360***
	(0.102)	(0.104)	(0.085)	(0.083)	(0.119)	(0.117)	(0.111)	(0.101)
Gender (Female)	−0.103	−0.097	−0.124	−0.067	0.073	0.125	0.254	0.324*
	(0.211)	(0.211)	(0.213)	(0.219)	(0.263)	(0.272)	(0.193)	(0.186)
Outage_Experience	−0.456***	−0.461***	−0.140	−0.187	−0.050	−0.093	0.151	0.094
	(0.151)	(0.154)	(0.158)	(0.155)	(0.218)	(0.219)	(0.193)	(0.197)
Household_Size	0.048	0.053	0.034	0.077	0.149*	0.187**	0.046	0.097
	(0.067)	(0.067)	(0.062)	(0.064)	(0.083)	(0.081)	(0.086)	(0.074)
Household_Income 2001–5000	0.366	0.369	0.935***	0.956***	−0.014	0.004	1.312***	1.336***
	(0.758)	(0.761)	(0.227)	(0.216)	(0.273)	(0.278)	(0.232)	(0.227)
Household_Income > 5000	0.633	0.626	0.843***	0.781***	−0.096	−0.152	1.188***	1.112***
	(0.738)	(0.743)	(0.250)	(0.241)	(0.298)	(0.308)	(0.286)	(0.282)
*Independent Variables*								
Feed-In	−0.008	0.023	0.146**	0.428***	−0.093	0.165	−0.094	0.253**
	(0.068)	(0.155)	(0.055)	(0.117)	(0.077)	(0.187)	(0.077)	(0.123)
Others-Orientation	0.282	0.381	0.416*	1.303***	0.375	1.185**	−0.016	1.073**
	(0.225)	(0.567)	(0.213)	(0.424)	(0.261)	(0.528)	(0.244)	(0.468)
Feed-In × Others-Orientation	–	−0.041	–	−0.367**	–	−0.335	–	−0.450***
	–	(0.181)	–	(0.143)	–	(0.213)	–	(0.161)
Help_Given	−0.042	−0.048	0.669***	0.620***	0.169	0.125	0.326*	0.267
	(0.190)	(0.195)	(0.194)	(0.194)	(0.195)	(0.200)	(0.191)	(0.190)
Help_Received	−0.567	−0.560	−0.140	−0.079	−0.368	−0.312	0.280	0.355
	(0.409)	(0.412)	(0.227)	(0.212)	(0.271)	(0.281)	(0.280)	(0.293)
Investment_Readiness	0.003	0.008	0.130	0.176**	0.083	0.124	0.246**	0.302***
	(0.079)	(0.084)	(0.082)	(0.081)	(0.097)	(0.099)	(0.096)	(0.099)
*Observations*	80	80	80	80	80	80	80	80
*R‑squared*	0.2231	0.2236	0.4131	0.4451	0.1767	0.2036	0.3840	0.4265

Robust standard errors are in parentheses

***p < 0.01, **p < 0.05, *p < 0.1

We theorize that structurally changing the payoffs of the electricity-sharing dilemma through increased *Feed-In *remuneration (Hypothesis 1a) increases participants’ willingness to share. As indicated in Table 2, only the estimate from Model 3 provides support for Hypothesis 1a (β = 0.15, *p* = 0.01), i.e. without accounting for people’s social value orientation (Messick and McClintock 1968), increasing feed-in remuneration only significantly increases their willingness to share towards friends and family.

To test Hypothesis 1b, the interaction of *Feed-In *remuneration and *Others-Orientation* is added in Models 2, 4, 6, and 8, respectively. These models therefore enable a separate analysis of the effects of increasing feed-in tariffs on more self- vs. more other-oriented individuals. This disentanglement provides very insightful results: For both recipient groups, friends and family in Model 4, as well as critical infrastructure in Model 8, increasing feed-in tariffs influences the willingness to share electricity among *self-oriented* respondents positively (β = 0.43, *p* < 0.01 for friends and family, and β = 0.25, *p* < 0.05 for critical infrastructure). Interestingly, this main effect is significantly reduced for *others-oriented* individuals as outlined by the negative interaction term in both models (β = −0.37, *p* < 0.05 for friends and family, and β = −0.45, *p* < 0.01 for critical infrastructure). As such, these results provide support for Hypothesis 1b for the friends and family as well as critical infrastructure groups.

To better understand the overall effect of increased feed-in tariffs on both groups, we calculated the partial (marginal) effect of *Feed-In* contingent on the level of *Others Orientation* holding all remaining variables at their sample means. For both stakeholder groups, the partial effect of *Feed-In *is positive and significant for *self-oriented* individuals (partial effect = 0.43, *p* < 0.01 for friends and family, and partial effect = 0.25, *p* < 0.05 for critical infrastructure). Put differently, for *self-oriented* individuals, increasing feed-in tariffs influences their willingness to share electricity positively. In contrast, performing the same analysis for *others-oriented* subjects leads to a non-significant partial effect that is close to zero (partial effect = 0.06, *p* > 0.10 for friends and family) or even negative (partial effect = −0.20, *p* < 0.05 for critical infrastructure). In other words, among *others-oriented *respondents, increasing feed-in tariffs will either lead to no change in sharing behaviour (in the case of friends and family), or even decrease the overall willingness to share electricity.

**Fig. 1:**
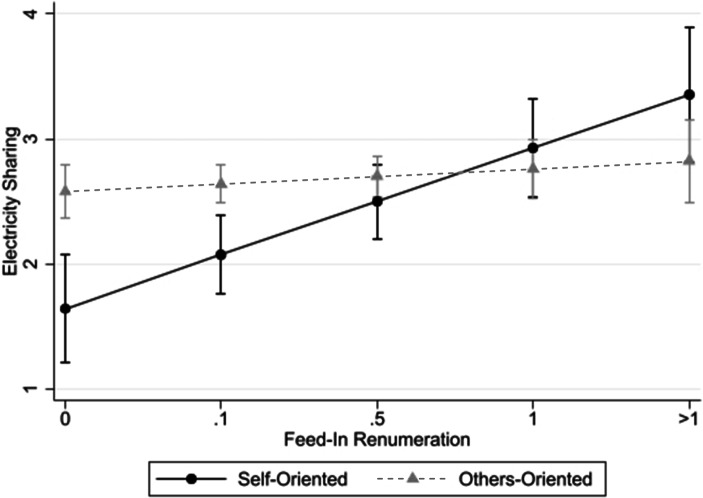
Effect of Feed-In Remuneration on Predicted Sharing Behaviour towards Friends & Family contingent on Social Value Orientation. (The figure shows the respective predicted sharing behaviour towards friends and family for different feed-in remuneration levels separately for self- and others-oriented respondents with 90% confidence intervals while holding all remaining variables at their sample means. The coefficients are taken from Model 4 of Table 2. The calculations were performed using STATA’s postestimation command “margins”)

**Fig. 2:**
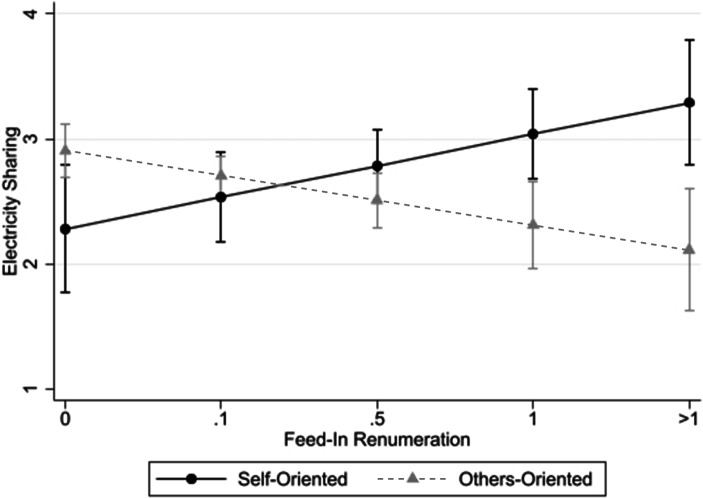
Effect of Feed-In Remuneration on Predicted Sharing Behaviour towards Critical Infrastructure contingent on Social Value Orientation. (The figure shows the respective predicted sharing behaviour towards critical infrastructure for different feed-in remuneration levels separately for self- and others-oriented respondents with 90% confidence intervals while holding all remaining variables at their sample means. The coefficients are taken from Model 8 of Table 2. The calculations were performed using STATA’s postestimation command “margins”)

To better visualize these results, we plotted the predicted sharing behaviour of different *Feed-In *remuneration levels separately for self- and others-oriented participants with 90% confidence intervals for both stakeholder groups in Figs. 1 and 2, respectively. The reported partial (marginal) effects are reflected in the slopes of the respective predicted regression lines. For the remaining stakeholders, i.e. neighbours as well as public infrastructure, the coefficients are not significant, implying that we can neither confirm nor reject Hypothesis 1b for these groups.

Concerning our empathy-elicited altruism Hypothesis 2, the coefficient for *Help_Given* is positive and significant in Models 3, 4, and 7, thus providing strong support for Hypothesis 2 concerning sharing behaviour towards friends and family (β = 0.67, *p* < 0.01 and β = 0.62, *p* < 0.01, respectively), and partial support concerning sharing behaviour towards critical infrastructure (β = 0.33, *p* < 0.10). No significant effect was found for the remaining two recipient groups. The coefficient for *Help_Received*, likewise, is not significant in any of the models. Consequently, we can neither confirm nor reject Hypothesis 3 for all investigated stakeholder groups.

Finally, the coefficient for *Investment_Readiness* is positive and significant in Models 4, 7, and 8, thereby providing moderate support for Hypothesis 4 on the influence of perceived threat for the sharing behaviour towards friends and family (β = 0.18, *p* < 0.05) as well as strong support for the sharing behaviour towards critical infrastructure (β = 0.25, *p* < 0.05 and β = 0.30, *p* < 0.01, respectively).

To validate the robustness of our results, we performed an additional outlier sensitivity analysis. Due to our prudent raw data cleaning, we reason that outliers remaining in our final dataset are likely to arise from natural variation in our sample instead of measurement errors (Aggarwal 2017; Wooldridge 2013). Sensitivity analysis with removal of these outliers is recommended for additionally confirming the robustness of the calculated results and providing transparency for the reader (Aguinis et al. 2010, 2013). Following this recommendation, we performed a sensitivity analysis with removal of these extreme values. Given our categorical data structure, we identified these extreme values as those categories with a low frequency of observations. Following best practice guidelines, we identified and removed the top and bottom categories with observation frequencies of less than 2.5% (Aguinis et al. 2013), which reduces our sample to 77 observations.

We then repeated the eight separate OLS regressions from our main analysis on this revised sample. Table 3 reports the new regression coefficients, their standard errors and their levels of significance for all variables. The results from this outlier sensitivity analysis are consistent with the findings from our main analysis, except that we also find weak significance for the moderating effect of others-orientation on the relationship between rewards and willingness to share electricity in Model 6 (Hypothesis 1b, β = −0.38, *p* < 0.10). The already only moderate effect of Help_Given (Hypothesis 2) in Model 7 from our main analysis is now insignificant (β = 0.33, *p* > 0.10). Given the weak, non-consistent significance level in both cases, we argue that both findings should be interpreted with caution, and refrain from inferring theoretical insights for these two effects in the subsequent discussion. For the remaining effects, however, we conclude that the consistent outcomes from this robustness analysis lend credence to the evidence from our main analysis.

**Table 3:** Regression Results Robustness Analysis

	Neighbours		Friends & Family		Public Infrastructure		Critical Infrastructure	
	Model 1	Model 2	Model 3	Model 4	Model 5	Model 6	Model 7	Model 8
	Electricity Sharing	Electricity Sharing	Electricity Sharing	Electricity Sharing	Electricity Sharing	Electricity Sharing	Electricity Sharing	Electricity Sharing
*Constant*	2.026*	1.783	0.878	−0.231	1.964*	0.825	1.950*	0.540
	(1.085)	(1.239)	(0.802)	(0.964)	(1.021)	(1.117)	(0.990)	(0.953)
*Control Variables*								
Age	0.020	0.029	−0.138	−0.095	−0.142	−0.098	−0.364**	−0.310**
	(0.137)	(0.142)	(0.134)	(0.132)	(0.170)	(0.165)	(0.168)	(0.151)
Gender (Female)	−0.167	−0.156	−0.113	−0.064	−0.049	0.001	0.222	0.284
	(0.218)	(0.217)	(0.228)	(0.232)	(0.255)	(0.262)	(0.198)	(0.186)
Outage_Experience	−0.423***	−0.432***	−0.143	−0.183	−0.061	−0.102	0.134	0.084
	(0.156)	(0.158)	(0.165)	(0.160)	(0.222)	(0.222)	(0.198)	(0.200)
Household_Size	0.046	0.057	0.038	0.089	0.141	0.193**	0.050	0.114
	(0.072)	(0.074)	(0.068)	(0.070)	(0.092)	(0.090)	(0.096)	(0.085)
Household_Income 2001–5000	0.340	0.341	0.933***	0.936***	−0.044	−0.041	1.295***	1.298***
	(0.766)	(0.767)	(0.236)	(0.225)	(0.272)	(0.275)	(0.239)	(0.229)
Household_Income > 5000	0.641	0.623	0.835***	0.756***	−0.131	−0.212	1.151***	1.051***
	(0.747)	(0.752)	(0.265)	(0.258)	(0.300)	(0.307)	(0.300)	(0.295)
*Independent Variables*								
Feed-In	−0.033	0.029	0.147**	0.434***	−0.108	0.187	−0.095	0.269**
	(0.076)	(0.158)	(0.060)	(0.120)	(0.085)	(0.196)	(0.082)	(0.126)
Others-Orientation	0.252	0.452	0.415*	1.331***	0.356	1.297**	−0.023	1.141**
	(0.236)	(0.590)	(0.220)	(0.437)	(0.265)	(0.560)	(0.251)	(0.481)
Feed-In × Others-Orientation	–	−0.084	–	−0.386**	–	−0.397*	–	−0.491***
	–	(0.196)	–	(0.152)	–	(0.228)	–	(0.175)
Help_Given	−0.067	−0.073	0.684***	0.654***	0.108	0.076	0.331	0.292
	(0.218)	(0.220)	(0.231)	(0.227)	(0.226)	(0.224)	(0.219)	(0.211)
Help_Received	−0.519	−0.506	−0.154	−0.095	−0.272	−0.212	0.293	0.368
	(0.412)	(0.413)	(0.245)	(0.230)	(0.275)	(0.283)	(0.293)	(0.300)
Investment_Readiness	0.021	0.033	0.131	0.188**	0.069	0.128	0.238**	0.311***
	(0.085)	(0.093)	(0.090)	(0.090)	(0.103)	(0.106)	(0.104)	(0.107)
*Observations*	77	77	77	77	77	77	77	77
*R‑squared*	0.2305	0.2325	0.4079	0.4418	0.1842	0.2227	0.3560	0.4067

Robust standard errors are in parentheses

***p < 0.01, **p < 0.05, *p < 0.1

## Discussion

The increasing number of natural disasters (Williams et al. 2017) as well as the rising share of renewable energies (Bialek 2020) raises concerns about the resilience of our energy networks (Lovins and Lovins 1982; Chaudry et al. 2009; Hirschl et al. 2018; Roege et al. 2014). At the same time, private PV systems become more and more popular, and our communities are in need of high volumes of energy since citizens consume more energy, e.g. through the increased use of electric vehicles. Against that background, experts call for approaches to investigate the use of this ever-growing number of residential PV systems and battery storage as dispersed energy sources to increase the system’s resilience against power outages (Kishita et al. 2017; Lovins and Lovins 1982; Perez et al. 2004). If technically prepared (Knodt et al. 2022), these households could contribute the electricity produced by their PV systems to (at least partially) maintain service for the society during disruption (Hirschl et al. 2018; Perez et al. 1997). However, this concept will only work if enough PV system owners choose to share their electricity and do not consume it themselves. This typical example of a social dilemma constitutes the essence of this study (Dawes 1980).

Motivated by the relevance and potential of this social dilemma for the resilience of our society, our goal was to develop an understanding of how people can be incentivized to behave cooperatively during a power outage. To this end, we developed a model of incentivizing cooperative action and postulated four main hypotheses on structural incentives and individual (motivational) factors (Messick and Brewer 1983). We then empirically tested the effect of the hypothesized motives on the sharing behaviour towards four different recipient groups and found the influences of the motives to strongly differ across these groups. Regarding the sharing behaviour towards friends and family as well as critical infrastructure, our results show that individual (intrinsic) motives are a much clearer predictor for cooperative behaviour than structural changes. More specifically, while empathy-elicited altruism (Batson 1991) and altruistic norms (Schwartz 1977) had a moderate to strong positive influence on the willingness to share electricity, the effect of increasing feed-in remuneration as a structural change interestingly depends on the subject’s level of social orientation (Messick and McClintock 1968; Van Lange et al. 2013). These effects shall be elaborated in more detail in the following.

Empathy-elicited altruism (Batson 1991) served as a strong predictor for the sharing behaviour towards friends and family. This coincides with prior research showing that the mechanisms of empathy are affected by processes of felt-similarity (Batson 1991; Bierhoff 2002). In fact, studies demonstrated that neural empathic reaction patterns in the brain differ depending on whether the victim is a friend or a stranger (Meyer et al. 2013), and that feelings of empathy are most pronounced when victims are similar (Krebs 1975; Bierhoff 2002). In line with this previous evidence, our results show that effects of empathy-elicited altruism increase the willingness to share electricity with related individuals (i.e. friends and family) in times of a power outage (Batson 1991).

Regarding altruistic norms, our results demonstrate that norms activated through perceived threat increase the sharing behaviour towards critical infrastructure strongly and towards friends and family moderately (Schwartz 1977). In other words, these outcomes show that the more individuals perceive the negative consequences of a power outage as a threat, the more their altruistic norm to help is activated, but only towards these two recipient groups. A plausible explanation for this finding could be found using the second condition of the norm activation theory: Reminiscing that this second condition refers to the feeling of responsibility (Schwartz 1977), these findings may imply that people feel most responsible for the critical infrastructure, and moderately responsible for friends and family. Therefore, we can infer from our results that altruistic norms only influence cooperative behaviour towards these two groups.

Concerning the influence of structural solutions (Messick and Brewer 1983) on the sharing behaviour towards friends and family as well as critical infrastructure, we found that increasing feed-in remuneration represents a double-edged sword: While it significantly *increases* cooperative behaviour among self-oriented individuals, it is ineffective (in case of friends and family) or even leads to a *decrease* (in the case of critical infrastructure) in the willingness to engage in such behaviour among those people with a strong level of others-orientation (Messick and McClintock 1968; Van Vugt 2001; Frey and Oberholzer-Gee 1997). One explanation for this result could be that – among these individuals—introducing such a reward scheme transforms a previously ethical issue into a business decision (Gneezy and Rustichini 2000; Tenbrunsel and Messick 1999), thereby replacing pre-existing intrinsic motives, which in turn either makes the rewards ineffective or even reduces the overall degree of cooperation (Bénabou and Tirole 2006; Frey 1994). This serves as a perfect example of the often-reported “crowding-out” effect, where intrinsic motivation is reduced or even fully replaced through psychological processes of impaired self-determination, impaired self-esteem, and impaired expression possibility (Frey and Oberholzer-Gee 1997; Frey 1997). Overall, these results underline that, at least concerning the cooperation behaviour towards related victims as well as critical infrastructure, individual (intrinsic) motives appear to be more effective for fostering electricity sharing than structural solutions and hence contribute to a higher societal resilience in times of power outage.

Concerning the sharing behaviour towards neighbours and public infrastructure, none of the theorized structural and motivational solutions was significant. The absence of incentive effects towards non-critical public infrastructure can be explained by a generally considerably lower willingness to share electricity even before offering any incentives. Possibly, citizens just do not believe that non-critical public infrastructure such as community centres require urgent help during times of adversity (Penner et al. 2005; Bierhoff 2002), and thus neither intrinsic nor reward-based motives are sufficiently relevant. Missing incentive effects towards neighbours may appear to be more surprising at first glance. However, we see several explanations for the absence of the hypothesized effects: First, incorporating the influence of relatedness on the effect size of empathy-elicited altruism (Batson 1991; Krebs 1975), it is reasonable that the effect of empathy-elicited altruism is not pronounced in the sharing behaviour towards neighbours, given that with the diminishing community identity, these are increasingly rarely perceived as socially close (Van Vugt 2002; Bierhoff 2002; Puddifoot 1995). In a similar manner, it further seems plausible that people feel less responsible for the well-being of their neighbours compared to that of their close relatives. Accepting this proposition implies that the second condition of Schwartz et al.’s (1977) norm activation theory is not met, thus explaining why the perceived threat cannot serve as a motive for cooperative behaviour. Finally, concerning the missing effect of rewards, we see one potential empirical explanation in the relative stability of sharing behaviour towards neighbours in our sample: Most respondents indicated their willingness to share parts of their electricity, with only a few respondents saying they would not share at all, and no participants were willing to contribute all their electricity. This suggests a relatively stable sharing behaviour towards neighbours that is less influenceable through structural changes (Van Lange et al. 2013). Overall, these results underline that the relevance of structural as well as individual motives differs significantly depending on the help recipient.

A final surprising result of our study is the lack of any effect motives of reciprocity (Trivers 1971). Despite the ubiquity of reciprocal norms in cultures globally (Penner et al. 2005; Schroeder et al. 1995; Komter 1996), our study could not identify any significant influence of reciprocal motives on the sharing-behaviour during a power outage. One theoretical factor inhibiting the effects of reciprocity could be the anonymity inherent in the analysed scenario: In our dilemma of electricity sharing, neither the public nor the help recipient can see the identity of the donor. As such, the fundamental assumption of direct reciprocity, i.e. the understanding to help explicitly those who have provided help in the past, is violated. Indeed, studies have shown that with increasing anonymity, reciprocal behaviour is reduced (Hardy and Van Vugt 2006; Fox and Guyer 1978; Axelrod and Hamilton 1984).

### Theoretical Implications

This study expands our understanding on how to achieve energy resilience (Kishita et al. 2017; Afgan and Veziroglu 2012). In recent decades, researchers unanimously reached the conclusion that our energy networks need to become more resilient (Roege et al. 2014; Gatto and Drago 2020), and that a favourable approach is the use of dispersed power generation (Lovins and Lovins 1982). It is obvious to integrate residential PV systems into this approach, using them as an alternative energy source in power outage situations (Kishita et al. 2017; Perez et al. 2004). Surprisingly though, research has, to the best of our knowledge, so far not investigated the willingness of PV system owners to fulfil that function. Still, this willingness is the necessary precondition to make this whole approach work. Through analysing structural and individual motives affecting this willingness, our work significantly advances our understanding on the feasibility of the PV approach for achieving energy resilience. On a broader perspective, Bichler et al. (2022) recently determined a general rarity of energy market studies in business research (Bichler et al. 2022). Since investigating incentives and resilience lies at the heart of organizational and thus business research (Williams et al. 2017), we argue that our work provides an important contribution in answering Bichler et al.’s (2022) “call to arms” for further research on energy networks in business literature (p. 3).

Second, our study also contributes to the social dilemma literature (Dawes 1980; Van Lange et al. 2013). Before this study, no work has previously analysed the degree of prosocial behaviour (Bierhoff 2002) in the social dilemma of electricity sharing during a power outage. Previous studies most similar to ours have focused on water instead of electricity supply (Van Vugt 2002, 2001). In line with previous common goods research on community effects (Ostrom 1990), they found that effective moderation of water consumption either requires adequate incentive structures or strong community ties (Van Vugt 2001). Our results show that these findings cannot simply be transferred to the social dilemma of electricity sharing. More precisely, structural interventions such as modified incentive structures (Messick and Brewer 1983) transpire to be a highly double-edged sword, with some positive effects among self-oriented individuals, but also pronounced crowding-out effects among others-oriented citizens (Hossain and Li 2013; Frey 1994).

At the same time, our results show significant effects from individual factors such as empathy-elicited altruism (Batson 1991) and altruistic norms (Schwartz 1977) driving altruistic behaviour towards related victims (Krebs 1975) as well as critical infrastructure. These outcomes outline the power and relevance of pre-existing prosocial motives for the sharing of electricity during power outages. Overall, our study delivers the important contribution that, even though the influence of motives significantly differs between recipient groups, the social dilemma of electricity sharing appears to be rather a game of individual (intrinsic) than structural strategies (Messick and Brewer 1983).

Prior research on resilience stresses the fact that resilient systems need to be able “to cope with shocks and to keep functioning in much the same kind of way” (Walker 2020, p. 1) and have the capacity to transform themselves. Following this postulation, our study provides specific insights on the motives of PV system owners to share their electric power and, in doing so, contribute to the energy system’s functioning and transformation during shocks. With these insights, our study further corroborates recent advances which postulated that for improving the resilience of our society, an integrated view of technical and societal resilience has to be applied which necessitates adaptive governance systems (Folke 2006).

### Practical Implications

In recent years, practice-oriented energy institutes increasingly insist on making our energy systems more resilient (Hirschl et al. 2018; Chaudry et al. 2009). Our work thus provides clear implications for policymakers. The power of pre-existing prosocial motives shown in our study underlines that, from a social and participatory perspective (Knodt et al. 2022), the approach of using residential PV systems in times of power outage serves as an immediate and feasible measure for quickly improving energy resilience. From a technical perspective however, the system requires some modifications, with grid-forming inverters to be installed (Knodt et al. 2022). We see the impetus for policymakers to take action here, for instance by subsidizing these installation costs to make them affordable and attractive for current and future PV system owners. In fact, subsidies were found to strongly increase the adoption rate of energy-saving home-devices such as solar panels, water meters, and roof insulation (Van Lange et al. 2013). As a positive side effect, we argue that this is likely to also further increase the willingness to share: If more individuals are *able* to share, the second condition of the norm activation model will be fulfilled for more individuals, thus activating their normative prosocial behaviour (Schwartz 1977). According to the classification about resilience in engineering systems by Wied et al. (2020), privately-owned PV systems are systems that are already in place but need to be adapted and improved to make them usable as a means of resilience in times of disturbance. A positive side effect of supporting and enabling private PV systems for outage events would be that the technical equipment can also be better used for stabilizing our energy system in non-crisis times. For instance, adapted inverters combined with storage solutions would enable the control when privately produced energy is contributed to the grid, e.g. feeding in energy when demand is high—a non-neglectable aspect as the current energy crisis proves (Holling 1996). Hence, an increased willingness of private PV system owners to share their energy would increase the overall communal energy system resilience.

However, policymakers should particularly be careful with the tempting idea to simply increase feed-in tariffs during outages. As outlined above, doing so may turn a social decision into a business decision particularly among others-oriented individuals (Gneezy and Rustichini 2000; Tenbrunsel and Messick 1999), thus having no or even a negative effect on the level of cooperation. Instead, the fostering of strong social ties seems more effective and should be followed by policymakers (Van Vugt 2002). More precisely, and complementing previous studies (Van Vugt 2001), our results show that both, the degree of relatedness and felt responsibility (Batson 1991; Schwartz 1977), serve as significant predictors for cooperative behaviour during a power outage. It therefore appears that resilient communities could be formed by measures that lead to the expansion of such feelings towards neighbours and thus beyond friends and family or critical infrastructure, as already found in this study. This approach relates to the establishment and fostering of a community identity (Puddifoot 1995). Such community identity building can be achieved by various measures, such as organized face-to-face group meetings among neighbours (Samuelson 1990; Lewin 1947) or the development of clear norms (Van Vugt 2002; Ostrom 1990). It is reasonable to assume that an active community fostering will be of even higher relevance in the future, given that the increased mobility and the associated heterogeneity has led to a strongly declining sense of community especially among Western cultures (Van Vugt 2002; Bierhoff 2002)—a fact that is plausibly linked to the lower level of prosocial acting towards neighbours found in this study. Without having tested community identity directly, our results lead us to carefully advise policymakers to implement measures for promoting community identity building (Samuelson 1990).

### Limitations and Future Research

As with all empirical work, our setting is subject to limitations. First, our work only covered a field study conducted in non-crisis times with a relatively small sample size. We are aware that our survey participants were put in a hypothetical setting since they were not studied during an ongoing power outage when answering the questionnaire. Their behaviour might be different when they are directly confronted with an outage. However, in order to derive measures how to incentivize people’s willingness to share their energy resources, one has to find out about their motives that induce sharing behaviour. Logically, such measures and the necessary technical adaptions require time to be prepared and implemented, which is why we were motivated to conduct our study despite this limitation. To inspire survey participants imagining their behaviour in a real outage setting, we included the question whether they already personally experienced a major power outage before, which 73% of respondents answered with ‘yes’. We also made them aware that further investments would be necessary to render their PV systems capable of doing so. Further, we argue that it is indispensable that in reality PV system owners need to give their consent to share their energy prior to an event of adversity. Policymakers need to find out more under which conditions people are willing to agree in order to know which incentives have to be set to make use of this resilience power of our energy network. Our results should therefore be considered under the scenario under which they were derived, particularly acknowledging that the context set might overestimate the level of cooperation (Bierhoff 2002). We therefore see a promising avenue for future research to complement our work with studies conducted in experiment-based settings, or, ideally, studies combining field research covering a larger sample with lab experiments to provide strong external validity and overcome design weaknesses of pure field research studies (Van Vugt 2001; Van Lange et al. 1992; Samuelson 1990). An obvious design for such experiments would be placing subjects in a simulated scenario of a long-lasting power outage and then observing their sharing behaviour.

A second limitation of our work is that our research design did not allow us to investigate the underlying mechanisms and processes at play. For instance, concerning the empathy-altruism hypothesis (Batson 1991), it would be interesting to directly measure the level of empathy and in turn its explicit influence on the decision to share electricity with the various stakeholder groups, and when exactly empathic processes are activated (Hodges and Wegner 1997). Regarding the altruistic norm activation (Schwartz 1977), an intriguing avenue for further research could be comparing the effect size of both conditions separately, i.e. are feelings of responsibility or perceived threat more important for altruistic action?

Third, our field research study design did also not allow us to measure visible direct reciprocity, i.e. the tendency to help those that have verifiably helped the subject (Trivers 1971; Hardy and Van Vugt 2006). Most likely driven by the anonymity inherent in our setting, we did not find any significant reciprocity effects. We are convinced that, given the ubiquity of reciprocal norms globally (Penner et al. 2005; Schroeder et al. 1995; Komter 1996), investigating these effects in a more experimental setup with iterated rounds allowing for visible reciprocal behaviour will provide interesting new insights. Such studies could for instance offer subjects the choice to not only share their electricity with anonymous stakeholder groups, but with specific recipients, for instance those that have previously offered help.

As indicated in our practical implications, we see one final intriguing research avenue for research measuring the effects of community identity on cooperative action during power outages (Puddifoot 1995). An ideal research setting for such a study would be a community with a proven strong level of community identity. Based on our results showing that feelings of relatedness and responsibility serve as a strong predictor of prosocial behaviour, it would be reasonable to expect that in such a community, mechanisms of empathy-elicited altruism (Batson 1991) as well as activated norms (Schwartz 1977) should also influence the electricity sharing towards neighbours, since these would then be considered as related and felt responsible for. Likewise, a generally higher level of cooperation behaviour also seems plausible. We leave it to future studies to disentangle these questions.

## Conclusion

To improve the resilience of our energy systems, we investigated the social feasibility of using residential PV systems as electricity source during a power outage. Our results demonstrate that the willingness to share electricity in such situations differs strongly depending on the recipient group: Concerning sharing with related recipients as well as critical infrastructure, empathy-elicited altruism and altruistic norms show a moderate to strong effect on the willingness to share. In contrast, structural approaches such as increased rewards turn out to be an ambiguous double-edged sword depending on the donor’s social value orientation. None of the tested motives demonstrates a significant effect on the sharing behaviour towards neighbours and public non-critical infrastructure. Overall, these findings imply that the social dilemma of electricity sharing is more a game of intrinsic motives than of structural rewards, which is of high practical relevance for policymakers when designing an energy system of increased resilience leveraging the voluntary sharing behaviour of private PV system owners. This underlines that there may be some truth to the old adage of true friends, with people manifestly behaving as friends indeed for their friends in need during a power outage.

## Footnotes

1 Defined as outages lasting more than 24 h.

2 The one observation of diverse gender was removed from the analysis for statistical reasons.
